# Nutritional and Protein Deficiencies in the Short Term following Both Gastric Bypass and Gastric Banding

**DOI:** 10.1371/journal.pone.0149588

**Published:** 2016-02-18

**Authors:** Judith Aron-Wisnewsky, Eric O Verger, Carine Bounaix, Maria Carlota Dao, Jean-Michel Oppert, Jean-Luc Bouillot, Jean-Marc Chevallier, Karine Clément

**Affiliations:** 1 Institute of Cardiometabolism and Nutrition, ICAN, Assistance Publique-Hôpitaux de Paris, Pitié-Salpêtrière hospital, Nutrition department, F-75013, Paris, France; 2 Sorbonne Universités, UPMC University Paris 06, UMR_S 1166 I, ICAN, Nutriomics team, F-75005, Paris, France; 3 INSERM, UMR_S U1166, NutriOmics team, F-75013, Paris, France; 4 Assistance Publique-Hôpitaux de Paris, Visceral surgery Department, Ambroise Paré Hospital, 92100 Boulogne-Billancourt, France; 5 Assistance Publique-Hôpitaux de Paris, Visceral surgery Department, Hopital Europeen Georges Pompidou, F-75015, Paris, France; Western University of Health Sciences, UNITED STATES

## Abstract

**Background:**

The number of morbidly obese patients undergoing bariatric surgery (BS) has increased dramatically in recent years. Therefore, monitoring food intake and its consequences in terms of nutritional status is necessary to prevent nutritional deficiencies. The aim of this study was to analyze the effect of food restriction on nutritional parameters in the short-term (≤3 months) period after BS in morbid obesity.

**Method:**

In a prospective study, we followed 22 obese women who underwent Roux-en-Y gastric bypass (GBP) or adjustable gastric banding (AGB) at baseline (T0) and 1 (T1) and 3 (T3) months after surgery. We evaluated food intake, nutrient adequacy and serum concentrations of vitamins and minerals known to be at risk for deficiency following BS.

**Results:**

Before surgery, we observed suboptimal food intakes, leading to a risk of micronutrient deficiencies. Serum analysis confirmed nutritional deficiencies for iron and thiamine for 27 and 23% of the patients, respectively. The drastic energy and food reduction seen in the short term led to very low probabilities of adequacy for nutrients equivalent across both surgeries. Serum analysis demonstrated a continuous decrease in prealbumin during the follow-up, indicating mild protein depletion in 21 and 57% of GBP patients and 50 and 63% of AGB patients, respectively, at T1 and T3. Regarding vitamins and minerals, systematic supplementation after GBP prevented most nutritional deficiencies. By contrast, AGB patients, for whom there is no systematic supplementation, developed such deficiencies.

**Conclusions:**

Our results suggest that cautious monitoring of protein intake after BS is mandatory. Furthermore, AGB patients might also benefit from systematic multivitamin and mineral supplementation at least in the short term.

## Introduction

Obesity is defined as an excess of body fat mass inducing adverse effects on health, which has become a worldwide epidemic, is also associated with nutritional deficiencies [1,2]. Indeed, although obese subjects display an excess caloric intake, they are prone to malnutrition as previously shown in obese individuals who had lower serum vitamin status than healthy lean controls matched for age and sex [3]. Studies evaluating morbidly obese patients before bariatric surgery (BS), also demonstrated subclinical serum protein depletion [4] as well as micronutrient deficiencies [5]. These findings may be the result of under consumption of foods such as fresh fruit, vegetables and lean meat during energy dense meals [6].

Therapeutic strategies to treat obesity are limited. Although effective, lifestyle intervention is both disappointing regarding the degree of weight loss [7] and its maintenance in the long term (≥6 months) [8]. Therefore, BS, which is currently recommended for patients with BMI above 40kg/m^2^ or above 35kg/m^2^ when associated with obesity-related diseases [9], has dramatically risen, reaching 468,000 interventions in 2013 worldwide (a 3.2-fold increase compared to 2003) [10]. BS enables major and sustainable weight loss as well as significant improvement of obesity related-diseases [11]. Multiple surgical procedures are available among which 60% are represented by Roux-en-Y gastric bypass (GBP) and adjustable gastric banding (AGB) [10]. Although decreasing in number of interventions, AGB remains the first choice in young obese patients, who become candidates for BS earlier in life [12,13]. Weight loss mechanisms after BS include food restriction due to gastric narrowing in all surgical procedures, and an added reduction in nutrient absorption due to proximal alimentary limb diversion in GBP which leads to a certain degree of malabsorption [14].

Although, food intake has already been evaluated at baseline and in the longer term post-BS [15–20], only macronutrient intake and a few micronutrients were analyzed such as iron or calcium [15]. Furthermore, the link between food intake reduction and its consequences on micronutrient status biomarkers were not systematically examined [15,16,20–22]. Other studies have only evaluated serum micronutrient status before and after BS, but not in link with food intake [5,23,24]. Moreover, only very few publications exist on food intake report in the short term after BS (≤3 months) and most without any associated measures of micronutrient serum concentration [25]. Finally, only two studies compared GBP to AGB in the long term [23,26] and none in the short term.

Due to poor absorption, GBP further exacerbates baseline nutritional deficiencies in the long term as acknowledged in multiple studies [5,15,24,27]. These observations have led to the systematic prescription of multivitamin and mineral supplements after GBP to be maintained over a lifetime [28,29]. Conversely, after AGB, supplementation is not routinely used but recommended only when mineral and vitamin deficiencies are detected [29].

We herein aimed to analyze food restriction effects on the nutritional adequacy of the diet, on macro- and micronutrient intake evolution, as well as their consequences in terms of bioclinical evolution and micronutrient serum concentration in the short term post-surgery comparing GBP and AGB, using the same methodology we previously published [30].

## Materials and Methods

### Patients

Consecutive patients recruited in this prospective non-randomized study were followed for care in the Obesity Unit of Pitié-Salpetrière Hospital, Institute of Cardiometabolism and Nutrition, ICAN, Paris, France. They were candidates for either AGB or GBP according to international BS guidelines [29] (i.e. body mass index (BMI) ≥40kg/m^2^, or ≥35kg/m^2^ with at least one obesity-related comorbidity). The choice of technique was based upon the choice of the patient and hospital multidisciplinary team discussion based on medical history, level of obesity, and obesity-related comorbidity. Weight stable patients were enrolled in this study from July 2012.

Medical history and clinical evaluation were obtained at baseline and during the follow-up at 1 (T1) and 3 months (T3) as described elsewhere [31]. Anthropometric parameters were estimated by whole-body fan-beam DXA scanning (Hologic Discovery W, software v12.6, 2; Hologic, Bedford, MA), as previously described [32]. Variables from DXA used in the analyses were total fat-free mass (FFM, in kg) and total fat mass (FM, in kg). Basal metabolic rate (BMR) was assessed with indirect calorimetry (Deltatrac II monitor, Datex Instrumentarium Corp., Helsinki, Finland) enabling the evaluation of underreporting of dietary intake [33]. Physical activity was briefly assessed using the validated Modifiable Activity Questionnaire (MAQ) to evaluate low, moderate or intense physical activity [34]. The ethics committees of the Hotel-Dieu hospital approved the clinical protocol (number AOM10285/P100111), which has been recorded on clinical trial website (NCT: NCT01454232). All patients gave their written informed consent prior to study inclusion.

### Dietary data and nutrient intakes

At baseline, T1, and T3, patients completed three 24h dietary records as described elsewhere [35], including 2 weekdays and 1 weekend day whenever possible. All foods and beverages consumed at breakfast, lunch, dinner or collation (occasion of consumption of light snacks) were recorded. Duration of each meal was recorded to evaluate food intake ingestion speed. Validated photographs enabled patients to estimate portion size for each reported food and beverage item [36]. Patients were also asked to indicate multivitamin and mineral supplement use, specifying the product name and amount, following our standardized nutritional deficiency prevention treatment described in [37]. This includes supplementation during 2 weeks before surgery of 25(OH)-vitamin-D3 (once 4× 100,000 IU), thiamine (250 mg/day), and vitamin B12 (250μg/day). Fifteen days post-GBP, multivitamin and mineral supplements including Azinc “Forme et vitalité”^®^ (two capsules per day, containing 800 μg vitamin A, 1.4 mg thiamine, 200 μg folate, 1 μg vitamin B12, 120 mg vitamin C, 200 IU vitamin D, 8 mg iron, and 15 mg zinc), iron (2×80 mg/day), 25(OH)-vitamin-D3 (800 IU/day), and calcium (1,000 mg/day) were started and continued for the first year in GBP procedures. Nutrient intakes from foods were calculated using an updated version of the French database CIQUAL 2008 [38] which included more than 3,400 different foods. Nutrient intakes from multivitamin and mineral supplements were calculated using nutrient profiles based on the product name. Ingested foods were categorized into four main food groups when possible: (i) fruit and vegetables, (ii) starchy foods, (iii) dairy products, and (iv) meat and fish. The food groups were defined according to the French National Nutrition and Health Program [39] and expressed in servings per day based on standard serving sizes [40].

### Nutrient adequacy of the diet

Nutrient intake adequacy for each patient was calculated using the PANDiet index [41]. Briefly, we calculated the probability of adequacy for each nutrient, ranging from 0 to 1, where 1 represents a 100% probability that the usual intake is adequate (i.e. it satisfies the requirement or is not excessive according to a reference value). According to this definition, the probabilities of adequacy were computed to obtain the Adequacy sub-score (the higher, the better means that the intakes satisfy the nutrient requirements) and the Moderation sub-score (the higher, the less chance the intakes are excessive). The PANDiet score is taken as the mean of the Adequacy and Moderation sub-scores, and ranges from 0 to 100; the higher the score, the better the nutrient adequacy of the diet. As reference values, we used French nutritional recommendations for healthy adults or European Union values when specific recommendations were lacking.

### Biochemical analyses

Blood samples were collected after an overnight fast to measure biochemical parameters using routine techniques as previously described [31]. Blood count, coagulation screen, and serum iron analysis were assessed. Prealbumin was assessed by immunoturbidimetry. Serum concentrations of 25(OH)-vitamin-D3 and parathyroid hormone (PTH) were measured by chemiluminescent assay (310600 Liaison XL Diasorin and 11972103 Modular E 170 Roche, respectively), vitamin B12 and folate were assessed using immunoanalysis ECL sandwich, and thiamine was assessed using high-performance liquid chromatographic method (HPLC) which was already validated in [3,42]. Vitamin and mineral deficiencies were defined as a result below the lower normal value given by the manufacturer [4]. Secondary hyperparathyroidism was defined as an elevated PTH, above the high normal laboratory value. All measurements were conducted at baseline, T1 and T3 (except for 25(OH)-vitamin-D3 and PTH which were measured at baseline and 6 months after surgery) as proposed by a recent recommendations [43].

### Statistical analyses

Continuous variables are presented as median and interquartile range (IQR) and frequencies as percentages. Mann-Whitney and paired Wilcoxon rank-sum tests were, respectively, used to compare continuous variables between surgical groups and time points. Chi square and McNemar’s tests were used to compare frequencies between surgical groups and time points, respectively. An overall α level of 5% was used for statistical tests following Holm-Bonferroni correction. All analyses were performed using Statistical Analysis Systems statistical software package version 9.3 (SAS Institute, Cary, NC, USA).

## Results

### Clinical Characteristics

Twenty-two women were included and completed the follow-up of this study (T1 and T3) with 14 undergoing GBP and 8 AGB. Importantly, before surgery, the two groups were similar regarding age, degree of obesity and body composition (Table 1). Likewise, the severity of obesity-related comorbidities was similar in the two groups, except for type-2 diabetes and glucose intolerance, which was significantly more prevalent in the GBP group (Table 1).

**Table 1:** Anthropometric parameters and clinical characteristics according the surgical models at baseline and 1 and 3 months.

	GBP (n = 14)			AGB (n = 8)		
	Baseline	1 month	3 months	Baseline	1 month	3 months
Age (years)	40.5 (31.0–45.0)	/	/	40.5 (32.0–43.5)	/	/
**Anthropometric parameters**						
Weight (kg)	119 (110–131)^c^	110 (100–119)^b^	100 (91–113)^a^	113 (110–118)^b^	109 (106–112)^a^^b^	103 (98–108)^a^
BMI (kg/m^2^)	46.3 (42.3–49.3)^c^	41.2 (38.9–43.7)^b^	38.2 (35.2–40.4)^a^	42.8 (42.4–43.8)^b^	40.6 (39.5–42.6)^a^^b^	39.1 (37.4–40.3)^a^
Weight loss (kg)	0.0 (0.0–0.0)^a^	11.1 (8.1–12.6)^b^	18.8 (15.7–28.0)^c^	0.0 (0.0–0.0)^a^	6.1 (3.7–7.4)ab*	11.6 (7.0–13.4)b*
Fat mass (%)	51.3^b^	50.5^b^	47.8^a^	49.8	49.6	47.7
Fat free mass (%)	46.7^a^	47.4^a^	49.2b	47.6	48.3	49.3
**Obesity related-diseases**						
Type-2 diabetes. N (%)	3 (21)	0 (0)	0 (0)	0 (0)*	0 (0)	0 (0)
Glucose intolerance. N (%)	4 (29)	2 (14)	1 (7)	0 (0)*	0 (0)	1 (13)
OSA. N (%)	6 (43)	6 (43)	3 (21)	4 (50)	1 (13)	2 (25)
Dyslipidemia. N (%)	13 (93)	13 (93)	13 (93)	7 (88)	7 (88)	7 (88)
HBP. N (%)	6 (43)	7 (50)	6 (43)	1 (13)	2 (25)	3 (38)

^a,b,c^ Median or percentage values within a row with unlike superscript letters were significantly different between time points for each surgical model, as tested by paired pairwise post hoc comparisons with Holm-Bonferroni correction or paired McNemar’s test.

*Significant differences between GBP and AGB. Glucose intolerance is defined as either fasting hyperglycemia (1 g/l≤G<1.26 g/l) or 6%≤HBA1c<6.5%; dyslipidemia is defined as a patient with treatment (statin or fibrate) or hypertriglyceridemia ≥1.5 g/l or hypoHDL<0.5 g/l; high blood pressure (HBP) is defined as a systolic blood pressure >140 mmHg and/or a diastolic blood pressure>90 mmHg or patients with an anti-hypertensive treatment; obstructive sleep apnea (OSA) is defined as an Apnea-Hypopnea Index >5/h with or without treatment.

As expected, BS induced significant weight loss in both surgical techniques (Table 1), with a greater effect after GBP compared to AGB. Interestingly, this weight loss mostly concerned FM, which occurred as early as 1 month after surgery. Conversely, FFM (in %) increased significantly, along the follow-up in the GBP group. Therefore, despite a moderate initial loss in FFM (in kg) and further stabilization, patients displayed a significant improvement in body composition (S1 Fig and Table 1).

### Food and Macronutrient Intakes

At baseline, no difference was observed for energy, food, or macronutrient intakes between the two groups (Table 2). Patients were weight stable for three months prior to their examination, and displayed low levels of physical activity. As detailed by Quesada et al. [44], we assessed the underreporting of energy intake based on the ratio between reported energy intake and indirect calorimetry measurement, and the calculation of the cutoff points. We found that 85.7% of the patients were considered as underreporters, as is frequently observed in bariatric surgery candidates [44]. There was no significant difference between the two surgical technic groups in terms of percentage of underreporters or intensity of underreporting.

**Table 2:** Energy, food, and macronutrient intakes according to the surgical models at baseline and 1 and 3 months.

	GBP (n = 14)			AGB (n = 8)		
	Baseline	1 month	3 months	Baseline	1 month	3 months
**Energy and food intakes**						
Energy intake. kcal/d	1427 (1194–1820)^b^	750 (500–1033)^a^	672 (509–1037)^a^	1248 (915–1581)	943 (712–1483)	958 (916–1256)
BMR. kcal/d	1937 (1734–2130)^b^	1919 (1687–1931)^b^	1762 (1575–1849)^a^	1823 (1710–1877)	1827 (1715–1859)	1771 (1721–1820)
Fruits and vegetables. serving/d	3.1 (2.0–3.5)^b^	0.4 (0.1–2.4)^a^	1.6 (0.4–2.8)^a^^b^	2.4 (1.4–4.1)	1.7 (1.3–2.6)	1.6 (1.0–1.8)
Starchy foods. serving/d	3.7 (2.5–4.2)^b^	0.7 (0.4–1.3)^a^	0.7 (0.3–1.2)^a^	3.0 (2.0–5.1)	1.4 (0.9–2.1)	1.4 (1.2–1.6)
Dairy products. serving/d	2.2 (1.3–3.1)	1.1 (0.7–1.9)	1.7 (0.7–2.7)	1.5 (1.2–2.2)	1.0 (0.6–1.2)	1.0 (0.3–2.2)
Meat and fish. serving/d	1.6 (1.0–1.7)^b^	0.5 (0.3–0.9)^a^	0.7 (0.4–0.9)^a^^b^	1.6 (0.7–2.2)	0.9 (0.7–1.1)	1.0 (0.8–2.0)
**Macronutrient intakes**						
Protein. g/d	67.8 (61.7–86.1)^b^	37.4 (22.4–43.6)^a^	35.5 (26.6–46.7)^a^	63.1 (47.0–83.1)	51.0 (42.6–55.6)*	49.1 (43.6–58.4)*
N (%) < 60*g/d*	2 (14)^a^	13 (93)^b^	14 (100)^b^	4 (50)	7 (88)	7 (88)
Protein. g/kg/d	0.58 (0.49–0.73)^b^	0.33 (0.20–0.40)^a^	0.37 (0.26–0.42)^a^	0.54 (0.43–0.75)	0.47 (0.39–0.57)*	0.51 (0.41–0.62)*
Total Lipid. %EI/d	31.4 (26.5–37.1)^a^	40.0 (35.5–42.5)^b^	33.6 (28.8–43.9)^a^^b^	34.6 (27.3–43.2)	39.0 (35.3–43.0)	43.0 (43.6–58.4)
SFA. %EI/d	12.4 (10.8–13.8)^a^	16.8 (15.4–17.7)^b^	15.1 (13.1–19.9)^a^^b^	12.4 (10.5–14.1)	15.5 (14.9–17.0)	15.8 (13.0–19.4)
PUFA. %EI/d	4.6 (4.2–6.4)	3.4 (2.9–6.2)	4.3 (3.8–5.5)	5.3 (4.6–5.9)	5.8 (4.1–6.5)	6.6 (5.1–7.7)
Total Carbohydrate. %EI/d	50.8 (43.6–53.4)^b^	42.7 (41.5–44.2)^a^	47.1 (35.1–53.2)^a^^b^	43.5 (37.9–51.7)	40.0 (35.3–45.1)	35.4 (32.3–39.8)

^a,b^ Median or percentage values within a row with unlike superscript letters were significantly different between time points for each surgical model, as tested by paired pairwise post hoc comparisons with Holm-Bonferroni correction or paired McNemar’s test.

*Significant differences between GBP and AGB. EI: energy intake. SFA: saturated fatty acids. PUFA: polyunsaturated fatty acids.

Table 2 displays energy, food and macronutrient intakes for each surgical model at baseline, T1 and T3. After GBP, starchy foods, meat and fish, and energy intakes decreased over time (significantly lower at T3 compared to baseline). Fruits and vegetables and dairy product intakes tended to decrease (T1) and then increase (T3). After AGB energy intakes and fruits and vegetables tended to decrease over time, whereas meat and fish, starchy foods and dairy products intakes tended to decrease at (T1) and then stabilize at (T3). Importantly, in both surgery groups, food intake caloric reduction involved all three meals without significant increase in collation energy intake, thus demonstrating that patients followed the prescribed dietary advice (S2 Fig). Furthermore, patients displayed a significant 2-fold decrease in food ingestion speed in both surgical models (15±6 vs. 6.3±3.2 kcal/min at baseline and T3 respectively) in agreement with clinical advice to chew their food slowly after surgery to improve food tolerance (S2 Fig) [45,46]. Likewise, vomiting or digestive discomfort was scarce in this cohort (data not shown).

A decrease in total protein intake was observed after both BS (Table 2). This decrease was drastic and significant after GBP as compared to AGB, resulting in significantly lower total protein intake in the GBP group at T1 and T3 (Table 2). Nevertheless, whatever the surgery group, the consumption of protein was below the recommended value of 60g/day for 88 to 100% of the patients post-BS (Table 2). After GBP, total fat and saturated fatty acids (SFA) significantly increased at T1 and tended to decrease at T3 whereas total carbohydrates significantly decreased at T1 and tended to increase at T3 (Table 2). After AGB, total fat and SFA tended to increase during the follow-up whereas total carbohydrates tended to decrease during the follow-up (Table 2). In both surgical models, carbohydrate consumption was mainly composed of sugars rather than starches (ratio sugars/starches around 60% of total ingested carbohydrate, S3 Fig).

### Nutrient Adequacy of the Diet

At baseline, neither the PANDiet scores nor the probabilities of nutrient adequacy differed between the two groups (Table 3). Low probabilities of adequacy for protein were observed in both groups as compared to the French adult population [41].

**Table 3:** Multivitamin and mineral supplementation, PANDiet scores, and probabilities of nutrient adequacy according to the surgical models at baseline and 1 and 3 months.

	GBP (n = 14)			AGB (n = 8)		
	Baseline	1 month	3 months	Baseline	1 month	3 months
**Supplementation. N (%)**	1 (7)^a^	11 (79)^b^	12 (86)^b^	0 (0)	1 (13)*	2 (25)*
**PANDiet**	63.7 (52.3–67.5)	70.1 (68.0–73.2)	69.5 (63.4–76.2)	58.2 (47.28–66.8)	53.9 (48.6–57.6)	50.5 (47.0–54.6)
**Moderation Sub-score**	75.0 (62.9–84.6)	74.3 (69.1–81.6)	76.2 (66.6–86.3)	73.5 (66.9–77.7)	77.6 (59.2–82.5)	65.4 (56.5–74.5)
Protein	0.85 (0.68–0.95)	0.99 (0.95–1.00)	0.98 (0.74–1.00)	0.88 (0.82–0.97)	1.00 (0.99–1.00)	0.95 (0.83–1.00)
Total Carbohydrate	1.00 (0.90–1.00)	1.00 (1.00–1.00)	1.00 (0.76–1.00)	1.00 (0.98–1.00)	1.00 (1.00–1.00)	1.00 (1.00–1.00)
Total Fat	1.00 (0.74–1.00)^b^	0.57 (0.29–0.89)^a^	0.83 (0.04–0.96)^a^^b^	0.90 (0.28–1.00)	0.67 (0.15–0.88)	0.18 (0.06–0.51)
SFA	0.43 (0.14–0.64)^b^	0.04 (0.00–0.15)^a^	0.31 (0.00–0.48)^a^^b^	0.43 (0.17–0.73)	0.12 (0.05–0.26)	0.08 (0.02–0.39)
Cholesterol	0.74 (0.48–0.97)	1.00 (0.98–1.00)	1.00 (0.76–1.00)	0.99 (0.711.00)	0.99 (0.70–1.00)	0.73 (0.47–0.95)
Sodium	0.64 (0.32–0.91)^a^	1.00 (0.99–1.00)^a^^b^	1.00 (0.93–1.00)^b^	0.72 (0.38–0.82)	0.97 (0.35–0.99)	0.99 (0.67–1.00)
**Adequacy Sub-score**	52.0 (36.9–63.7)	65.0 (58.7–69.9)	71.5 (63.4–74.8)	40.6 (29.7–55.8)	36.9 (24.8–50.1)	36.1 (28.7–44.9)
Protein	0.47 (0.45–0.51)^b^	0.33 (0.22–0.42)^a^^b^	0.33 (0.18–0.46)^a^	0.45 (0.43–0.52)	0.35 (0.29–0.43)	0.45 (0.34–0.49)
Total Carbohydrate	0.93 (0.47–0.99)	0.27 (0.16–0.50)	0.67 (0.00–0.97)	0.56 (0.02–0.96)	0.12 (0.00–0.54)	0.02 (0.00–0.12)
Total Fat	0.63 (0.03–0.86)	1.00 (0.95–1.00)	0.75 (0.33–1.00)	0.90 (0.28–1.00)	1.00 (0.82–1.00)	1.00 (1.00–1.00)
PUFA	0.35 (0.19–0.75)	0.09 (0.01–0.89)	0.33 (0.10–0.60)	0.66 (0.30–0.74)	0.71 (0.28–0.89)	0.79 (0.44–0.95)
Fibre	0.01 (0.00–0.08)^b^	0.00 (0.00–0.00)^a^^b^	0.00 (0.00–0.00)^a^	0.03 (0.00–0.23)	0.00 (0.00–0.04)	0.00 (0.00–0.00)
Vitamin A	0.86 (0.70–0.95)	1.00 (0.90–1.00)	1.00 (0.99–1.00)	0.67 (0.26–0.92)	0.73 (0.03–0.95)	0.28 (0.00–0.86)
Thiamine	0.53 (0.34–0.86)	1.00 (0.92–1.00)	1.00 (1.00–1.00)	0.52 (0.23–0.80)	0.46 (0.06–0.84)	0.22 (0.05–0.72)
Riboflavin	0.90 (0.47–0.95)	1.00 (1.00–1.00)	1.00 (1.00–1.00)	0.73 (0.24–0.89)	0.39 (0.13–0.64)	0.60 (0.24–0.83)
Niacin	0.95 (0.79–1.00)	1.00 (0.92–1.00)	1.00 (1.00–1.00)	0.93 (0.44–1.00)	0.83 (0.36–0.98)	0.79 (0.53–0.95)
Vitamin B-6	0.67 (0.36–0.94)	1.00 (0.80–1.00)	1.00 (1.00–1.00)	0.23 (0.02–0.76)	0.19 (0.00–0.57)	0.12 (0.01–0.51)
Folate	0.64 (0.25–0.89)	0.78 (0.61–0.97)	0.95 (0.76–0.99)	0.61 (0.21–0.84)	0.22 (0.11–0.61)	0.26 (0.05–0.40)
Vitamin B-12	0.89 (0.79–0.96)	0.73 (0.41–0.85)	0.80 (0.66–0.98)	0.46 (0.10–0.81)	0.73 (0.32–0.84)	0.81 (0.72–0.92)
Vitamin C	0.36 (0.13–0.65)^a^	1.00 (0.71–1.00)^a^^b^	1.00 (0.99–1.00)^b^	0.14 (0.00–0.60)	0.09 (0.03–0.30)	0.25 (0.10–0.64)
Vitamin D	0.01 (0.00–0.03)^a^	0.97 (0.60–1.00)^a^^b^	0.99 (0.89–1.00)^b^	0.00 (0.00–0.00)	0.00 (0.00–0.01)	0.01 (0.00–0.05)
Vitamin E	0.06 (0.00–0.18)^a^	0.93 (0.43–1.00)^a^^b^	0.98 (0.79–0.99)^b^	0.01 (0.00–0.37)	0.00 (0.00–0.22)	0.05 (0.01–0.71)
Calcium	0.53 (0.12–0.97)	0.96 (0.38–1.00)	1.00 (0.98–1.00)	0.55 (0.08–0.70)	0.08 (0.02–0.36)	0.14 (0.04–0.39)
Magnesium	0.00 (0.00–0.00)	0.00 (0.00–0.00)	0.00 (0.00–0.00)	0.00 (0.00–0.00)	0.00 (0.00–0.00)	0.00 (0.00–0.00)
Zinc	0.34 (0.22–0.79)	1.00 (0.85–1.00)	1.00 (1.00–1.00)	0.30 (0.07–0.77)	0.06 (0.05–0.17)	0.08 (0.03–0.45)
Phosphorus	0.99 (0.91–1.00)	0.54 (0.01–0.89)	0.39 (0.03–0.94)	0.99 (0.65–1.00)	0.96 (0.62–0.99)	0.85 (0.77–0.93)
Potassium	0.49 (0.32–0.73)^b^	0.00 (0.00–0.04)^a^	0.01 (0.00–0.08)^a^	0.18 (0.05–0.76)	0.08 (0.01–0.43)	0.02 (0.00–0.08)
Iron	0.60 (0.45–0.93)	1.00 (0.85–1.00)	1.00 (0.75–1.00)	0.35 (0.15–0.80)	0.30 (0.12–0.84)	0.60 (0.15–0.81)

^a,b^ Median or percentage values within a row with unlike superscript letters were significantly different between time points for each surgical model, as tested by paired pairwise post hoc comparisons with Holm-Bonferroni correction or paired McNemar’s test.

*Significant differences between GBP and AGB. SFA: saturated fatty acids. PUFA: polyunsaturated fatty acids.

After GBP, the percentage of patients taking the prescribed systematic multivitamin and mineral supplements (as seen in the food diary reports) significantly increased, from baseline to T3: 7 versus 86% for GBP as expected by the recommendations (Table 3). Due to this supplementation, the global nutrient adequacy of the diet did not drop but rather stabilized along the follow-up (PANDiet score and Adequacy sub-score were not significantly different at all-time points). Furthermore, the probabilities of adequacy for vitamins C, D and E were improved (Table 3). Of note, when the global nutrient adequacy of the diet was calculated without taking into account the prescribed supplementation, the PANDiet score and the Adequacy sub-score significantly decreased at T1 and T3 as compared to baseline. Such decrease was explained by significantly lower probabilities of adequacy for protein, fiber, zinc, potassium and iron, but also by non-significant trends of lower probabilities of adequacy for the other micronutrients (S1 Table). Importantly, since the prescribed supplementation neither contains protein, fiber, nor phosphorus, lower probabilities of adequacy for these nutrients were observed at T3 compared to baseline (Table 3).

After AGB, only patients with deficiency were prescribed with multivitamin and mineral supplements. Therefore, the percentage of patients taking these supplementations was lower than the GBP group reaching 13 and 25% at T1 and T3, respectively (Table 3). As a result, the decrease of the global nutrient adequacy of the diet was similar to that of patients from GBP when not taking into account the prescribed supplementation (trends not reaching significance, Table 3). There was no significant difference of the nutrient adequacy of the diet between the two surgical models at baseline, T1 and T3, with and without taking into account the prescribed supplementation.

### Nutritional Deficiencies

At baseline, none of the metabolic and nutritional parameters were different between the two groups, except for the concentrations of 25(OH) vitamin D3 and erythrocyte folate which were lower in the AGB group (Table 4). As expected in severe obesity, more than 70% of patients from both groups presented 25(OH)-vitamin-D3 deficiency as seen by serum concentrations below 30 ng/ml (Table 4) with subsequent secondary hyperparathyroidism in 45% of the patients, showing major deficiency in this population. Furthermore, 27% of the patients displayed authentic iron deficiency, as seen by low level of ferritin (below the normal range (N) for premenopausal women, 30≤N≤300μg/l) which translated into reduced erythrocyte globular volume in 18% of the patients (mean volume 76.5±1.3μm^3^). These results led to the prescription of iron supplementation to treat this biologically proven deficiency. Medical and morphological examination did not display any signs of bleeding. Similarly, 23% of the patients displayed thiamine deficiency (as seen with thiamine concentrations below the normal range; [126–250 nmol/l]), which was consistent with a low probability of adequacy for thiamine (Table 3).

**Table 4:** Metabolic and nutritional parameters according the surgical models at baseline and 1 and 3 months.

	GBP (n = 14)			AGB (n = 8)		
	Baseline	1 month	3 months	Baseline	1 month	3 months
**Hemoglobin (g/dl)**	13.1 (12.8–13.5)	13.3 (12.5–13.5)	13.3 (12.7–13.8)	13.2 (12.4–14.2)	12.4 (12.2–13.5)	12.8 (12.1–13.6)
<12 g/dl N(%)	3 (21)	1 (7)	2 (14)	2 (14)	1 (13)	1 (13)
**Ferritin (μg/l)**	59 (26–95)^a^	88.5 (50–141)^b^	77 (54–155)^b^	87 (18–126)	59 (45–99)	59 (34–110)
<30 μg/l N(%)	4 (28)	1 (7)	1 (7)	2 (25)	1 (13)	1 (13)
**Iron (μmol/l)**	12.0 (11.0–16.0)	11.0 (9.0–13.0)	14.5 (11.0–16.0)	15.0 (11.0–16.0)	12.0 (11.0–15.0)	12.5 (8.0–16.0)
<9 μmol/l N (%)	2 (14)	3 (21)	1 (7)	0 (0)	1 (13)	3 (38)
**Albumin (g/l)**	35.5 (34.0–38.5)^a^	37.5 (35.0–40.0)^b^	36.0 (35.0–38.0)^a^	36.0 (34.0–39.0)	37.0 (36.0–40.0)	38.5 (34.5–40.0)
<37 g/l N(%)	8 (57)	6 (42)	8 (57)	4 (50)	2 (25)	3 (38)
**Prealbumin (g/l)**	0.27 (0.24–0.30)^c^	0.24 (0.20–0.25)^b^	0.19 (0.17–0.23)^a^	0.22 (0.21–0.24)b	0.18 (0.17–0.24)^a^	0.19 (0.16–0.23)^a^
<0.2 g/L N(%)	0 (0)^a^	3 (21)^b^	8 (57)^c^	1 (13)^a^	4 (50)^b^	5 (63)^b^
**Calcium (mmol/l)**	2.27 (2.22–2.34)	2.34 (2.30–2.37)	2.27 (2.25–2.33)	2.24 (2.18–2.34)	2.32 (2.24–2.37)	2.37 (2.26–2.40)
**25(OH) vitamin D3 (ng/ml)**	19.0 (13.0–24.0)			10.0 (6.0–18.0)*		
<30 ng/ml N(%)	10 (71)			7 (88)		
**Parathyroid hormone (pg/ml)**	41.1 (33.3–54.0)			54.6 (43.5–77.5)		
>45 pg/ml N(%)	5 (35)			5 (63)		
**Thiamine (nmol/l)**	158 (132–182)			132 (113–168)		
<126 nmol/l N(%)	2 (14)			3 (38)		
**Erythrocyte folate (nmol/l)**	1342 (923–1650)			929 (864–1025)*		
**Serum folate (nmol/l)**	14.7 (12.0–23.6)			12.7 (11.0–15.2)		
<7 nmol/l N(%)	3 (21)			5 (63)		
**Vitamin B-12 (pmol/l)**	294 (222–392)			289 (208–618)		
<140 pmol/l N(%)	1 (7)			0 (0)		

^a,b,c^ Median or percentage values within a row with unlike superscript letters were significantly different between time points for each surgical model, as tested by paired pairwise post hoc comparisons with Holm-Bonferroni correction or paired McNemar’s test

*Significant differences between GBP and AGB. Normal ranges are as follows: hemoglobin 12–17 g/dl; ferritin 30–300 μg/l; iron 9–27 μmol/l; albumin 37–50 g/l; prealbumin 0.2–0.35 g/l; calcium 2.1–2.65 mmol/l; 25(OH)-vitamin-D3 30–100 ng/ml; thiamine 126–250 nmol/l; serum folate 7–39.5 nmol/l, vitamin B12 140–490 pmol/l.

After BS, as a consequence of low protein intake, prealbumin concentration significantly decreased during the follow-up, reaching the same level after both surgeries at T3 (Table 4). Subsequently, at T3, around 60% of the patients from both groups presented mild protein depletion as shown by prealbumin concentration below the normal range of 0.2 g/l. More than 40% of patients from both groups presented risk of mild protein malnutrition as shown by albumin concentration below the normal value of 37 g/l (Table 4). We checked that our patients did not display acute inflammation. Indeed, baseline mean CRP was within the low grade inflammation range (8.5 ±4.7 mg/l), thus not affecting prealbumin values [47]. This low grade inflammation significantly decreased in the short term with a mean CRP value of 3.9±3.2 (*P* = 0.001).

**Fig 1:**
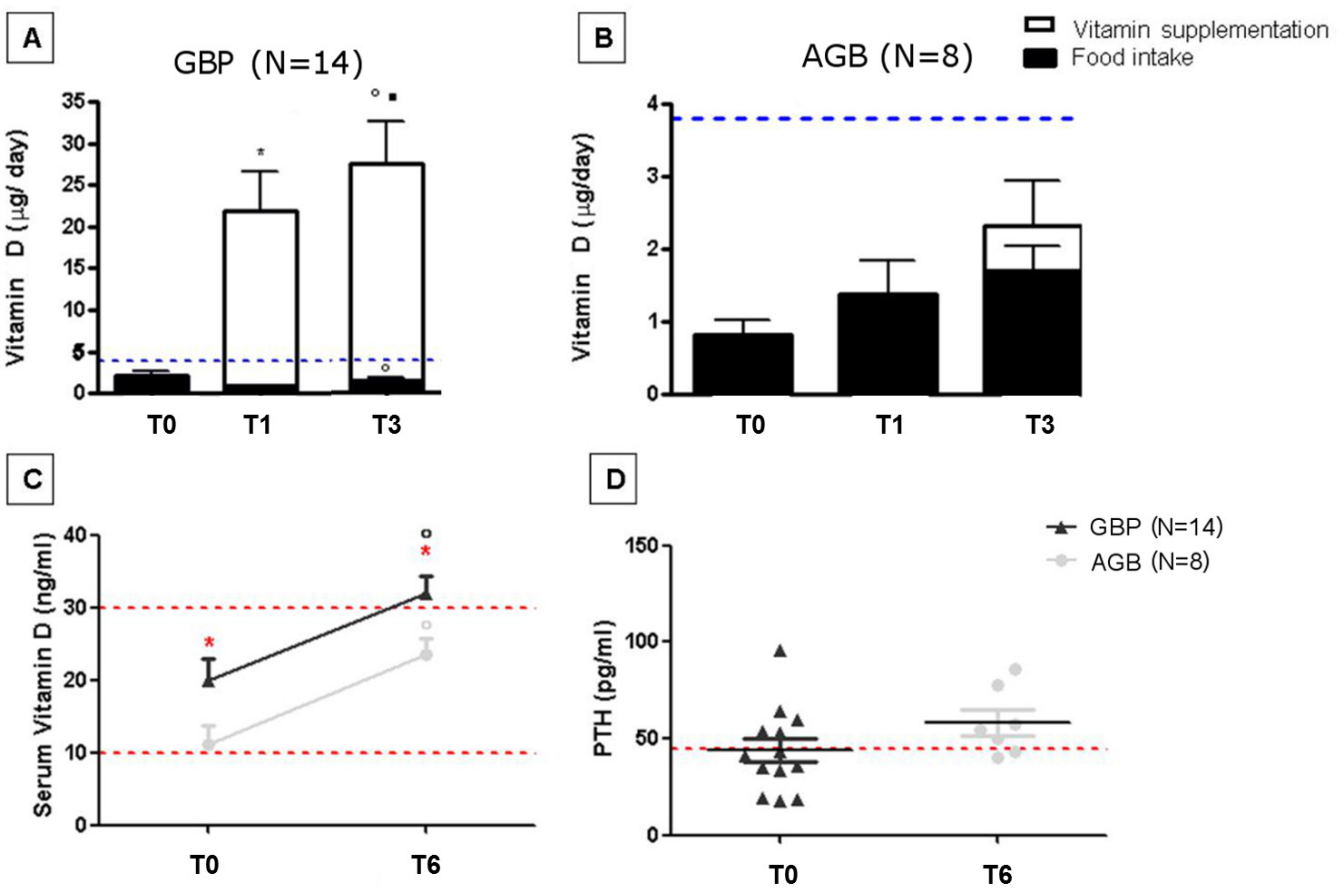
Vitamin D intake and phosphocalcic metabolism in patients undergoing GBP and AGB at baseline and during the follow-up. Results are expressed as means ± SEMs; significant differences if p<0.05. * represents significant differences between T0 and T1. ■ represents significant differences between T1 and T3.° represents significant differences between T0 and T3. * in red represents significant differences between AGB and GBP. **A**: Vitamin D food and supplement intake in GBP patients at baseline and during the follow-up. **B**: Vitamin D food and supplement intake in patients operated from AGB surgery at baseline and during the follow-up (*Black bars represent vitamin D intake from food and open bars those from vitamin supplementation given orally*. *Blue line represents the daily recommended intake)*. **C**: Serum vitamin D concentrations at baseline and 6 months after the surgery. Dark grey represents bypass patients and light grey AGB patients (*Lower red line represents the value below which vitamin deficiency is defined*, *the higher line represent the value below which vitamin D insufficiency is defined)* **D**: Parathormone serum concentration at baseline in both groups: dark grey for GBP patients and light grey AGB patients (*Red line represents the normal value above which secondary hyperthyroidism is defined)*.

Patients who underwent GBP are systematically supplemented with 25(OH)-vitamin-D3, thus this nutrient intake was increased largely above the recommended daily intake (7 fold) (Fig 1A). However, no toxicity was seen since calcium serum levels remained within the normal range during the follow-up (Table 4). Following the European recommendations [48], we did not measure this concentration at T3. However, 25(OH)-vitamin-D3 serum concentrations evaluated at 6 months displayed that this large supplementation only resulted in vitamin D normalization after GBP but not after AGB group (Fig 1C). These results are consistent with the malabsorption component of GBP. Conversely, since patients who underwent AGB are supplemented with vitamin D only if a deficiency is seen upon biological evaluation, the probability of adequacy for vitamin D was not improved (Table 3) and the 25(OH)-vitamin-D3 serum concentrations remained below the normal range 6 months after the surgery (Fig 1B).

**Fig 2:**
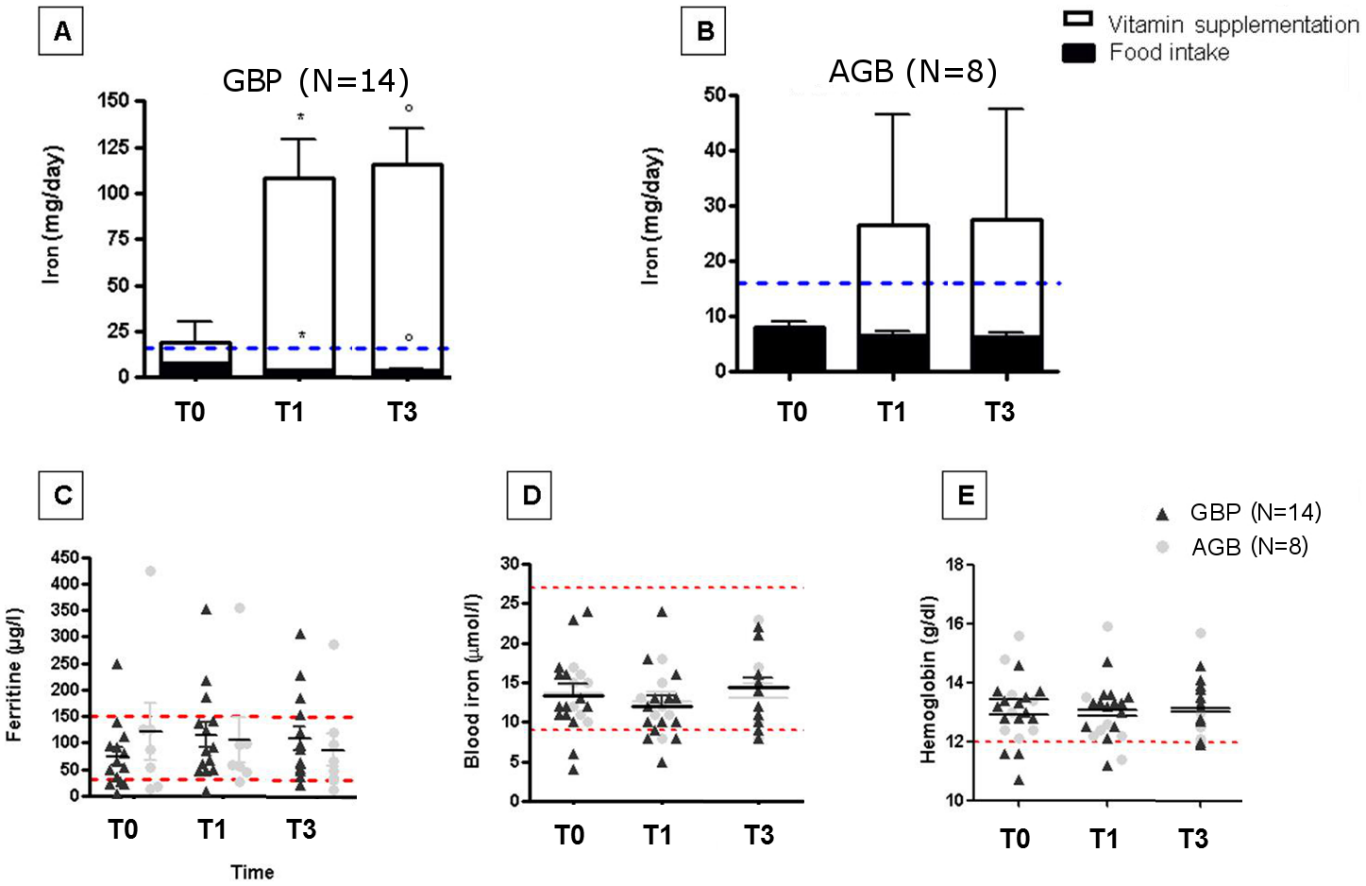
Iron intake and serum concentration of iron, ferritin and hemoglobin in patients undergoing GBP and AGB at baseline and during the follow-up. Results are expressed as means ± SEMs; significant differences if p<0.05. * represents significant differences between T0 and T1. ■ represents significant differences between T1 and T3.° represents significant differences between T0 and T3. * in red represents significant differences between AGB and GBP. **A**: Iron food intake in patients operated from bypass surgery at baseline and during the follow-up. **B**: Iron food intake in patients operated from AGB surgery at baseline and during the follow-up (*Black bars represent iron intake from food and open bars those from mineral supplementation given orally*. *Blue line represents the recommended intake per day to cover people’s need)*. **C**: Serum ferritin concentrations at baseline and 1 and 3 months after the surgery. Dark grey represents bypass patients and light grey AGB patients (*Lower red line represents the value below which iron deficiency is defined*, *the higher line represent the upper limit for normal ferritin levels)* **D**: Serum iron concentrations at baseline and 1 and 3 months after the surgery. Dark grey represents bypass patients and light grey AGB patients (*Lower red line represents the value below which defines iron deficiency*, *the higher line represent the limit for toxicity)*. **E**: Serum hemoglobin at baseline and 1 and 3 months after the surgery. Dark grey represents bypass patients and light grey AGB patients (*Lower red line represents cutoff for anemia)*.

Regarding iron, the systematic supplementation prescribed to GBP patients induced an increase in the probability of adequacy which reached the level of a reference population (Table 3). The measured iron daily intake reached 6 fold that of the recommended daily intake (Fig 2A), however remaining below toxic values as indicated by normal blood iron and ferritin level (Fig 2C and 2D). This supplementation effectively treated patients who presented discrete anemia at baseline (Fig 2E). Conversely, since patients who underwent AGB are supplemented with iron only if needed, the probability of adequacy for iron was not improved (Table 3). After AGB, 2 patients needed iron supplementation (same doses as GBP), among whom one demonstrated persistent iron deficiency suggesting that the number of patients with iron deficiency increased along the follow-up (Fig 2C and 2D). Of note, none of these patients developed anemia at the time of follow-up.

## Discussion

To the best of our knowledge, this is one of the first reports that studies the relationship between food intake, nutrient adequacy of the diet and nutritional biological parameters measured before and in the short term (≤3 months) after GBP and AGB. In this study where the patients had similar clinical characteristics at baseline (except for type-2 diabetes and glucose intolerance prevalence), our main findings are: (i) protein intake significantly decreases after both surgeries, inducing mild protein depletion in 59% of all the patients at T3, (ii) AGB is not harmless, since it significantly reduces food consumption, leading to biologically proven vitamin and mineral deficiencies. This suggests that a systematic multivitamin and mineral supplementation could be required at least in the short term, (iii) systematic multivitamin and mineral supplementation after GBP seems to prevent these early nutritional deficiencies.

Literature concerning food intake on the short term post-BS is scarce and does not combine measures of micronutrient serum concentrations concomitantly with enlarged dietary assessment [25]. Studies have either addressed food consumption [15,16,21,49], or have evaluated panels of serum micronutrient level before and after surgery [5,23,24] but few did both [50]. Only two studies compared GBP and AGB in the long term but no data was presented in the first months post-surgery [23,26].

The novelty of this work lies in 24h dietary records that we used, which enabled us to monitor and quantify a large panel of macronutrients and micronutrients in relation to systemic concentration measurements. Indeed, although previous studies have evaluated food intake at baseline and in the long term (≥6 months) after GBP, using various methods such as FFQ [16–18], 24h recall [15,19] or dietician interview [20], only macronutrients and few micronutrients were quantified. As shown in our work, drastic food reduction does not provide the recommended levels of some micronutrients [15,19,25,49], particularly for iron, thus inducing serum proven, objective iron deficiency and anemia [15]. Overall, these results reinforce the importance of precisely monitoring BS candidates and correcting their deficiencies prior to surgery, as already suggested [3–5,30,51,52]. If after GBP, it is mandatory to relate the reduced food intake to its biological consequences, our results highlights a new important finding: its important relevance in purely restrictive procedures such as AGB in these at-risk populations. Besides, using the same approach, we have previously also displayed nutritional deficiency after sleeve gastrectomy (SG), a primarily restrictive procedure [30].

At baseline, our population already displayed food intake below healthy recommendations as seen with low consumption of fruits and vegetables (less than 3 versus more than 5 servings per day) and dairy products (2 versus 3 servings per day). Meat and fish, and starchy foods consumptions met the recommendations [53]. These food patterns could partly explain the low probabilities of micronutrients adequacy of our patients, thus increasing their risk of nutritional deficiencies compared to the general population as previously observed [3,5]. Of note, most of the nutrient intakes have been underestimated due to the important level of underreporting at baseline, as is frequently observed in obese patients. This might have led to misestimate of the PANDiet score of the patients [54]. Had we taken into account this underreporting, the Moderation sub-score would have been lower and the Adequacy sub-score higher. More importantly, since patients reported correctly their food intake post-BS (comparison between 24h recall and dietician interview: data not shown), we might even have underestimated the decreases of food and nutrient intakes described below.

After surgery, the decrease in meat and fish intakes, linked to small intake of dairy products, worsened the probability of adequacy for proteins, iron and zinc. Likewise the reduction of starchy food intake, linked to small ingestion of fruits and vegetables, deteriorated the probability of adequacy for fiber and soluble vitamins, especially thiamine. These results were expected due to the nature of bariatric surgery, which forces patients to restrict their global food intake. Vegetables and red meat often represent the most decreased food intake since they are digested with difficulty [55]. In addition to this nutrient sparse diet, we also observed that patients tended to consume more sugars than starches. This could be due to easier food tolerance and ingestion at least shortly after surgery. However, clinicians should be cautious of this dietary pattern. Indeed GBP is frequently associated with delayed post-prandial hypoglycemia [56], which is caused by an increased consumption of sugars, but can be easily alleviated with diet modifications (namely increasing starch intake) [57].

Multivitamin and mineral supplement intake in patients who underwent GBP not only prevented a decrease of the global nutrient adequacy of the diet, but also improved the probabilities of adequacy for some vitamins. However, only relying on the use of supplements will not prevent low intakes of protein and fiber. Indeed, all of our GBP patients did not meet the recommendation to consume 60g of protein per/day [43]. Only one patient from the AGB group succeeded in meeting this recommendation at T3. This result is in accordance with what we previously observed in an independent cohort of patients undergoing GBP or SG [30]. Similarly, Andreu et al found that 45% of patients had a daily protein intake below 60 g/day 4 months after BS [58]. In our previously published study comparing GBP and sleeve, the inability to meet this recommendation resulted in 57% of GBP patients exhibiting mild protein depletion at T3. Most importantly, we also previously demonstrated that this low protein intake in the short term was not normalized in the longer term at T12 thus inducing around 50% of mild protein depletion in both SG and GBP at T12 [30]. Ensuring a sufficient protein intake has been shown to improve the efficiency of BS in terms of weight loss and improvement in body composition in both the short term (4 months) [59] and longer term (>12 months) [59,60]. Importantly, we also show that the important risk of mild protein depletion is not limited to GBP, but also occurs after AGB, which is new to our knowledge. This is of special clinical importance since this surgical technique, although decreasing in total number, is currently being more widely proposed to younger obese patients including those of pediatric age [61,62]. Adequate protein intake after BS is of utmost importance to prevent patients from experiencing adverse long-term effects on growth and body composition in this young population.

Regarding thiamine, we display objective deficiency in both surgical models in agreement with a previous observation [63]. This finding supports our proposition to systematically add oral thiamine supplementation 15 days before any type of BS and a systematic multivitamin containing thiamine after GBP [37]. Thiamine requirement increases after BS, especially but not exclusively after procedures leading to malabsorption. Since AGB patients also displayed thiamine deficiency, our results suggest that specific supplementation could be systematically proposed before surgery. Thiamine deficiency after BS is rather common and can lead to serious neurologic or cardiac diseases in both techniques [64,65]. This risk is heightened with surgical complications or simple vomiting which is very frequent after AGB and band adjustments [66]. However, supplementing only patients showing clinical signs of thiamine deficiency would prevent the treatment of patients with subclinical deficiency. We recommend the systematic vitamin supplementation of AGB patients at least in the short term after surgery for two reasons. First, we observed definite low probability of adequacy for different vitamins and minerals. Secondly, we identified proven thiamine deficiency, for which serum assessment is costly and time consuming.

Although carefully performed, our study has evaluated a small number of patients, which might have prevented us from finding significant changes in other nutrient intakes before and after surgery, especially in the AGB group. However, despite the small number of patients, we still identified significant nutrient deficiencies, particularly for protein. Longer follow-up of this cohort, on diet evaluation and its biological consequences, is needed since most studies report an initial drastic caloric reduction at 3 months and a partial recovery thereafter, with total caloric intake after surgery remaining significantly lower than baseline values [15,19,21].

In conclusion, this study combines a thorough quantitative evaluation of food intakes in terms of macro and more importantly micronutrients, adequacy score and serum concentrations of vitamins and minerals. It provides important clinical findings about deficiencies in the short term after both GPB and AGB which, if untreated, could have negative implications in the long term. Obesity and its surgical treatment, especially purely restrictive interventions are becoming more prevalent in adults and also in younger populations. Since nutritional deficiency can be neglected, we strongly suggest monitoring protein intakes, both before and after the surgery, and promoting consumption of protein-rich foods among a balanced diet, with added specific protein supplementation if needed in both GPB and AGB. Finally, our results suggest that candidates to AGB might benefit from systematic multivitamin and mineral supplementation.

## Supporting Information

S1 Fig Changes in body composition (kg fat mass and fat free mass) in the GBP and AGB groups at baseline and along the follow-up. Results are expressed as means ± SDs; significant differences if p<0.05. * represents significant differences between T0 and T1. ■ represents significant differences between T1 and T3.° represents significant differences between T0 and T3.
(TIF)

S2 Fig Daily food intake per meal (upper panel), and speed of food ingestion per meal in the GBP group (lower left panel) and in the AGB group (lower right panel). Results are expressed as mean ± SEMs; significant differences if p<0.05. * represents significant differences between T0 and T1.° represents significant differences between T0 and T3. * in red represents significant differences between GBP and AGB.
(TIF)

S3 Fig Sugar or starch intake expressed as percent of total carbohydrate intake at baseline and along the follow-up. Results are expressed as mean ± SEMs; significant differences if p<0.05. * represents significant differences between T0 and T1.° represents significant differences between T0 and T3.
(TIF)

S1 Table PANDiet scores and probabilities of nutrient adequacy according to the surgical models at baseline, 1 month and 3 months (calculated from foods only). ^a,b^ Median or percentage values within a row with unlike superscript letters were significantly different between time points for each surgical model, as tested by paired pairwise post hoc comparisons with Holm-Bonferroni correction or paired McNemar’s test. SFA: saturated fatty acids. PUFA: polyunsaturated fatty acids.
(DOCX)
