# Supplementary figures and images for: Nutritional and Protein Deficiencies in the Short Term following Both Gastric Bypass and Gastric Banding
Source: PLoS One. 2016 Feb 18;11(2):e0149588. doi: 10.1371/journal.pone.0149588 (PMC4758752; doi:10.1371/journal.pone.0149588)

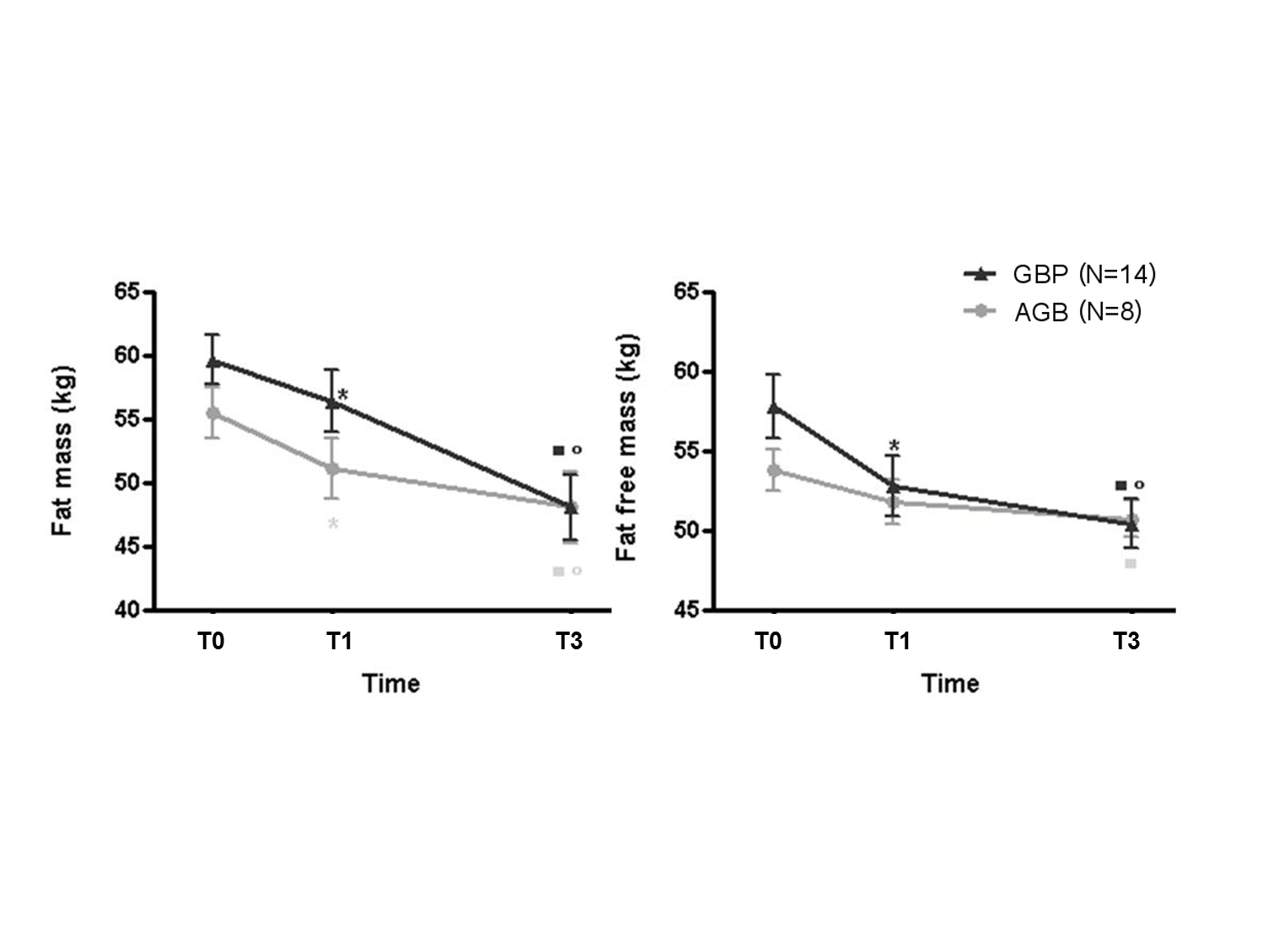

Supplement: S1 Fig — Results are expressed as means ± SDs; significant differences if p<0.05. * represents significant differences between T0 and T1. ■ represents significant differences between T1 and T3.° represents significant differences between T0 and T3. (TIF) [file pone.0149588.s001.tif]

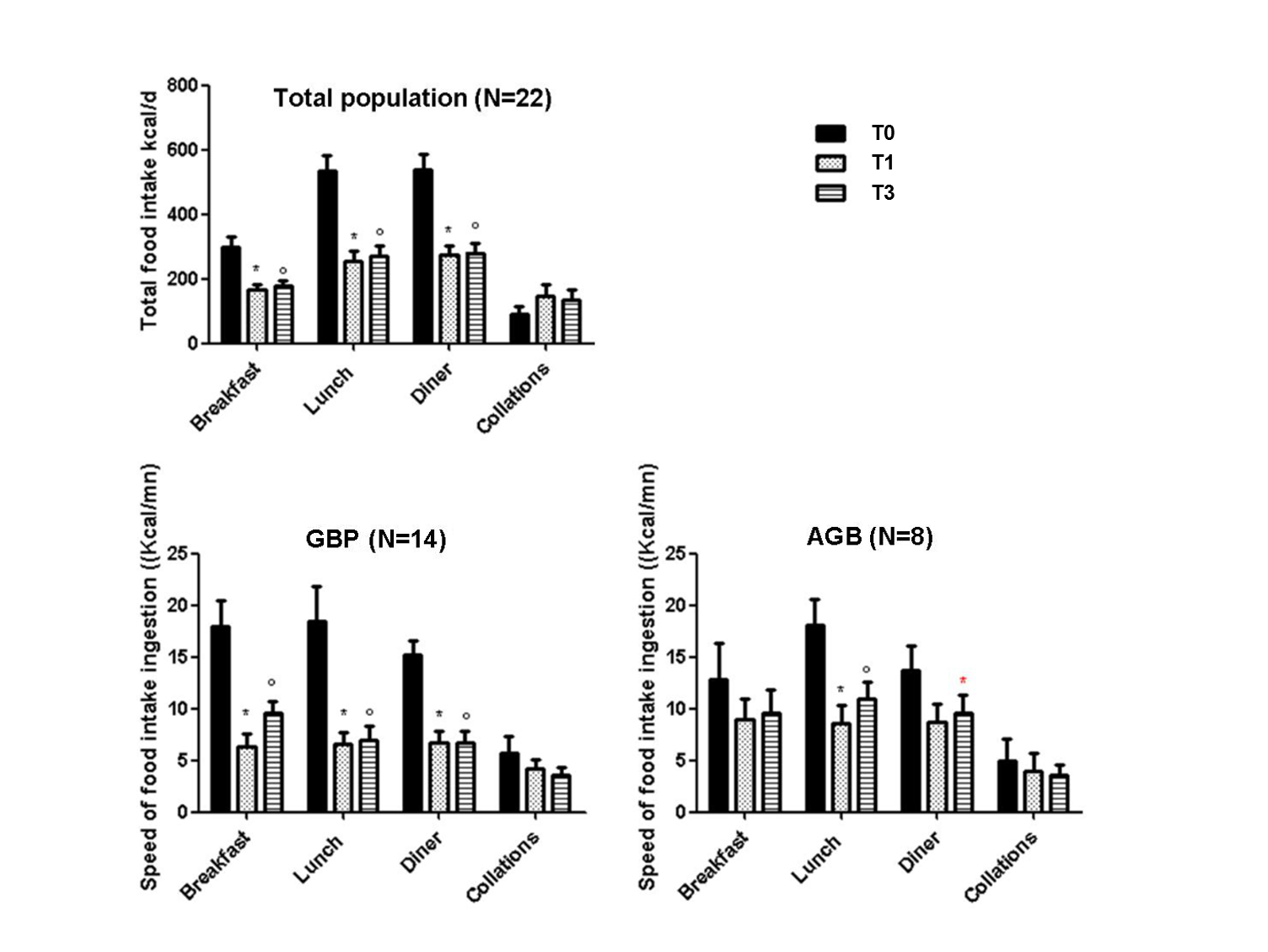

Supplement: S2 Fig — Results are expressed as mean ± SEMs; significant differences if p<0.05. * represents significant differences between T0 and T1.° represents significant differences between T0 and T3. * in red represents significant differences between GBP and AGB. (TIF) [file pone.0149588.s002.tif]

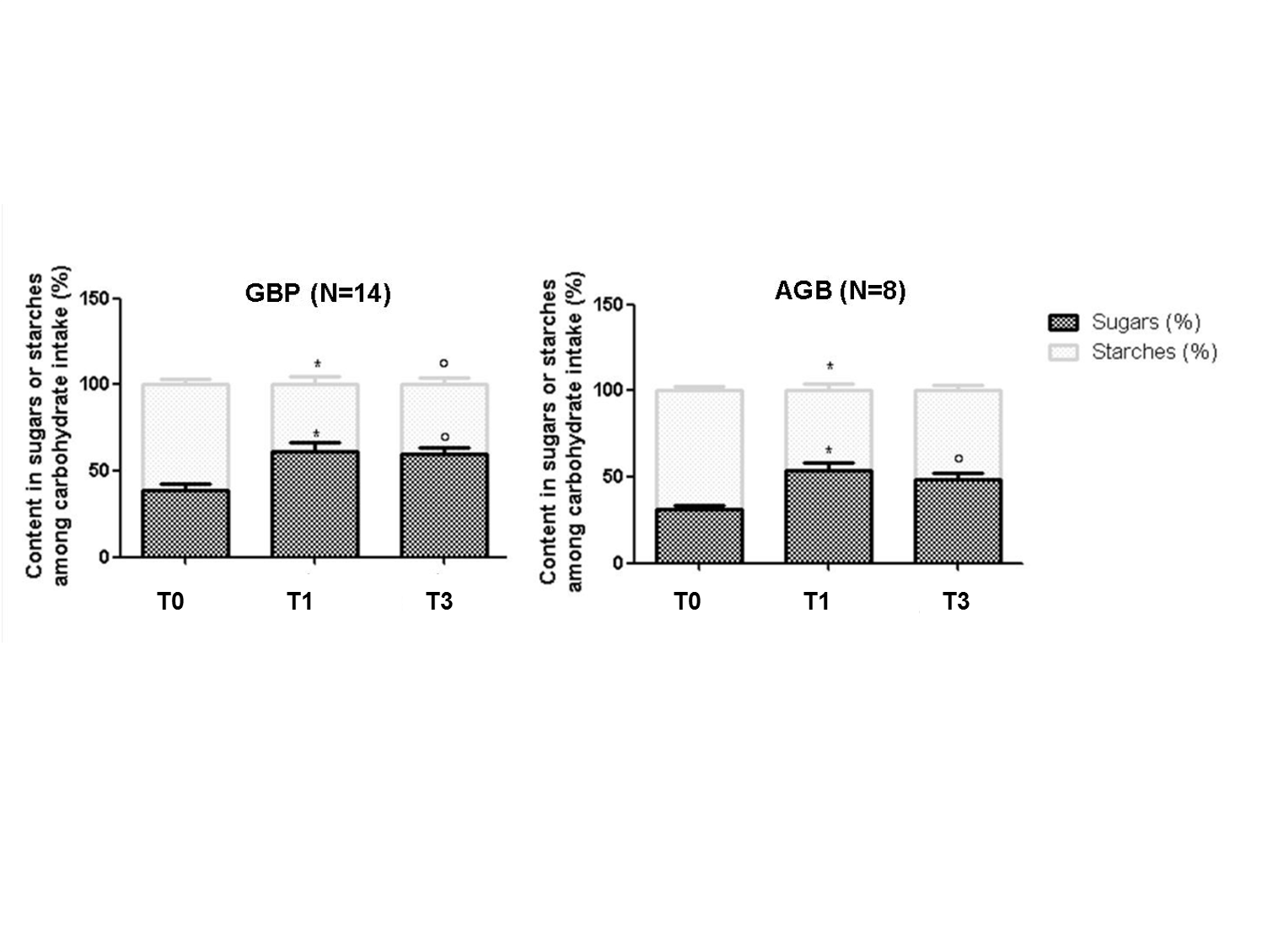

Supplement: S3 Fig — Results are expressed as mean ± SEMs; significant differences if p<0.05. * represents significant differences between T0 and T1.° represents significant differences between T0 and T3. (TIF) [file pone.0149588.s003.tif]
