# Supplementary material for: Nutritional and Protein Deficiencies in the Short Term following Both Gastric Bypass and Gastric Banding
Source: PLoS One. 2016 Feb 18;11(2):e0149588. doi: 10.1371/journal.pone.0149588 (PMC4758752; doi:10.1371/journal.pone.0149588)
Supplement: S1 Table — a,b Median or percentage values within a row with unlike superscript letters were significantly different between time points for each surgical model, as tested by paired pairwise post hoc comparisons with Holm-Bonferroni correction or paired McNemar’s test. SFA: saturated fatty acids. PUFA: polyunsaturated fatty acids. (DOCX) [file pone.0149588.s004.docx]

**Supplemental Data**

S1 Table**.** PANDiet scores and probabilities of nutrient adequacy according to the surgical models at baseline, 1 month and 3 months (calculated from foods only).

|  | GBP (n=14) | | | AGB (n=8) | | |
| --- | --- | --- | --- | --- | --- | --- |
|  | Baseline | 1 month | 3 months | Baseline | 1 month | 3 months |
| **PANDiet** | 63.7 (52.3-67.5)b | 47.6 (44.3-50.0)a | 48.2 (43.6-53.7)a | 58.2 (47.28-66.8) | 53.2(48.6-57.6) | 48.5 (46.9-52.8) |
| **Moderation Sub-score** | 75.0 (62.9-84.6) | 74.3 (69.1-81.6) | 76.2 (66.6-86.3) | 73.5 (66.9-77.7) | 77.6 (59.2-82.5) | 67.3 (56.5-74.5) |
| Protein | 0.85 (0.68-0.95) | 0.99 (0.95-1.00) | 0.98 (0.74-1.00) | 0.88 (0.82-0.97) | 1.00 (0.99-1.00) | 0.95 (0.83-1.00) |
| Total Carbohydrate | 1.00 (0.90-1.00) | 1.00 (1.00-1.00) | 1.00 (0.76-1.00) | 1.00 (0.98-1.00) | 1.00 (1.00-1.00) | 1.00 (1.00-1.00) |
| Total Fat | 1.00 (0.74-1.00)b | 0.57 (0.29-0.89)a | 0.83 (0.04-0.96)ab | 0.90 (0.28-1.00) | 0.67 (0.15-0.88) | 0.18 (0.06-0.51) |
| SFA | 0.43 (0.14-0.64)b | 0.04 (0.00-0.15)a | 0.31 (0.00-0.48)ab | 0.43 (0.17-0.73) | 0.12 (0.05-0.26) | 0.08 (0.02-0.39) |
| Cholesterol | 0.74 (0.48-0.97) | 1.00 (0.98-1.00) | 1.00 (0.76-1.00) | 0.99 (0.711.00) | 0.99 (0.70-1.00) | 0.73 (0.47-0.95) |
| Sodium | 0.64 (0.32-0.91)a | 1.00 (0.99-1.00)ab | 1.00 (0.93-1.00)b | 0.72 (0.38-0.82) | 0.97 (0.35-0.99) | 0.99 (0.67-1.00) |
| **Adequacy Sub-score** | 52.0 (34.6-63.7)b | 17.9 (12.0-28.2)a | 16.0 (12.4-40.1)a | 40.6 (29.7-55.8) | 36.9 (24.8-49.7) | 30.0 (28.2-39.1) |
| Protein | 0.47 (0.45-0.51)b | 0.33 (0.22-0.42)ab | 0.33 (0.18-0.46)a | 0.45 (0.43-0.52) | 0.35 (0.29-0.43) | 0.45 (0.34-0.49) |
| Total Carbohydrate | 0.93 (0.47-0.99) | 0.27 (0.16-0.50) | 0.67 (0.00-0.97) | 0.56 (0.02-0.96) | 0.12 (0.00-0.54) | 0.02 (0.00-0.12) |
| Total Fat | 0.63 (0.03-0.86) | 1.00 (0.95-1.00) | 0.75 (0.33-1.00) | 0.90 (0.28-1.00) | 1.00 (0.82-1.00) | 1.00 (1.00-1.00) |
| PUFA | 0.35 (0.19-0.75) | 0.09 (0.01-0.89) | 0.33 (0.10-0.60) | 0.66 (0.30-0.74) | 0.71 (0.28-0.89) | 0.79 (0.44-0.95) |
| Fibre | 0.01 (0.00-0.08)b | 0.00 (0.00-0.00)ab | 0.00 (0.00-0.00)a | 0.03 (0.00-0.23) | 0.00 (0.00-0.04) | 0.00 (0.00-0.00) |
| Vitamin A | 0.86 (0.70-0.96) | 0.39 (0.07-0.75) | 0.09 (0.00-0.27) | 0.67 (0.26-0.92) | 0.73 (0.03-0.95) | 0.28 (0.00-0.78) |
| Thiamine | 0.53 (0.34-0.86) | 0.00 (0.00-0.49) | 0.06 (0.00-0.66) | 0.52 (0.23-0.80) | 0.46 (0.06-0.84) | 0.13 (0.01-0.49) |
| Riboflavin | 0.90 (0.47-0.95) | 0.01 (0.00-0.11) | 0.04 (0.00-0.50) | 0.73 (0.24-0.89) | 0.39 (0.13-0.64) | 0.38 (0.13-0.77) |
| Niacin | 0.95 (0.79-1.00) | 0.25 (0.00-0.79) | 0.24 (0.01-0.74) | 0.93 (0.44-1.00) | 0.83 (0.36-0.98) | 0.74 (0.53-0.87) |
| Vitamin B-6 | 0.67 (0.36-0.94) | 0.00 (0.00-0.05) | 0.00 (0.00-0.31) | 0.23 (0.02-0.76) | 0.19 (0.00-0.57) | 0.03 (0.00-0.31) |
| Folate | 0.64 (0.25-0.89) | 0.01 (0.00-0.23) | 0.01 (0.00-0.46) | 0.61 (0.21-0.84) | 0.22 (0.11-0.61) | 0.23 (0.05-0.33) |
| Vitamin B-12 | 0.89 (0.79-0.96) | 0.35 (0.00-0.69) | 0.37 (0.09-0.72) | 0.46 (0.10-0.81) | 0.73 (0.32-0.84) | 0.81 (0.72-0.90) |
| Vitamin C | 0.36 (0.13-0.65) | 0.01 (0.00-0.43) | 0.02 (0.00-0.26) | 0.14 (0.00-0.60) | 0.09 (0.03-0.30) | 0.10 (0.00-0.25) |
| Vitamin D | 0.01 (0.00-0.03) | 0.00 (0.00-0.00) | 0.00 (0.00-0.11) | 0.00 (0.00-0.00) | 0.00 (0.00-0.01) | 0.01 (0.00-0.05) |
| Vitamin E | 0.06 (0.00-0.18) | 0.00 (0.00-0.00) | 0.00 (0.00-0.06) | 0.01 (0.00-0.37) | 0.00 (0.00-0.22) | 0.03 (0.00-0.37) |
| Calcium | 0.53 (0.12-0.97) | 0.05 (0.00-0.07) | 0.05 (0.00-0.70) | 0.55 (0.08-0.70) | 0.08 (0.02-0.36) | 0.14 (0.04-0.39) |
| Magnesium | 0.00 (0.00-0.00) | 0.00 (0.00-0.00) | 0.00 (0.00-0.00) | 0.00 (0.00-0.00) | 0.00 (0.00-0.00) | 0.00 (0.00-0.00) |
| Zinc | 0.34 (0.22-0.79)b | 0.02 (0.00-0.08)ab | 0.00 (0.00-0.02)a | 0.30 (0.07-0.77) | 0.06 (0.05-0.17) | 0.06 (0.03-0.17) |
| Phosphorus | 0.99 (0.91-1.00) | 0.54 (0.01-0.89) | 0.39 (0.03-0.94) | 0.99 (0.65-1.00) | 0.96 (0.62-0.99) | 0.85 (0.77-0.93) |
| Potassium | 0.49 (0.32-0.73)b | 0.00 (0.00-0.04)a | 0.01 (0.00-0.08)a | 0.18 (0.05-0.76) | 0.08 (0.01-0.43) | 0.02 (0.00-0.08) |
| Iron | 0.55 (0.35-0.85)b | 0.00 (0.00-0.15)a | 0.00 (0.00-0.25)a | 0.35 (0.15-0.80) | 0.25 (0.12-0.60) | 0.35 (0.12-0.60) |

^a,b^ Median or percentage values within a row with unlike superscript letters were significantly different between time points for each surgical model, as tested by paired pairwise post hoc comparisons with Holm-Bonferroni correction or paired McNemar’s test. SFA: saturated fatty acids. PUFA: polyunsaturated fatty acids.
